# Comparative Performance of GPT-5.1, Claude Sonnet 4.5, Gemini 2.0 Flash, and DeepSeek-R1 on Oral and Maxillofacial Radiology Questions From the Turkish Dental Specialisation Examination

**DOI:** 10.7759/cureus.114773

**Published:** 2026-08-19

**Authors:** Gaye Keser, Burcu Celen, Filiz Namdar Peki̇ner

**Affiliations:** 1 Department of Oral Medicine and Radiology, Marmara University, Istanbul, TUR; 2 Department of Oral and Maxillofacial Radiology, Marmara University, Istanbul, TUR

**Keywords:** board exam, deep learning artificial intelligence, large language models (llms), specialty doctor, student education

## Abstract

Introduction: Rapid advances in artificial intelligence (AI) and large language models (LLMs) have increased interest in their use in dental education and assessment. This study evaluated the accuracy of GPT-5.1, Claude Sonnet 4.5, Gemini 2.0 Flash, and DeepSeek-R1 on Turkish Dental Specialisation Exam (DUS) questions in oral and maxillofacial radiology.

Methods: A total of 132 Turkish DUS questions from 2012 to 2021 were obtained from a publicly accessible question bank, including 126 theoretical and six image-based items. Each question had five options and one correct answer. Questions covered radiologic physics, radiation safety, dental anatomy, pathology classification, imaging instrumentation, and visual interpretation of panoramic, periapical, cone beam computed tomography (CBCT), and clinical images. All models received identical prompts, and image-based items were submitted through the image-upload functionality available in the respective web interfaces. Responses were scored against the official answer key by two oral and maxillofacial radiologists. Statistical analyses included Fisher’s exact test, Cochran’s Q test, and Bonferroni-corrected McNemar tests. An exploratory secondary analysis evaluated session-to-session variation using a model-based performance index.

Results: GPT-5.1 answered all 132 questions correctly (100%), compared with Claude Sonnet 4.5 (119/132; 90.2%), Gemini 2.0 Flash (111/132; 84.1%), and DeepSeek-R1 (108/132; 81.8%). Overall performance differed significantly among the models (p < 0.001), and GPT-5.1 significantly outperformed each of the other three models after Bonferroni correction. On the six image-based questions, accuracy was 100% for GPT-5.1, 83.3% for Claude Sonnet 4.5, 50.0% for Gemini 2.0 Flash, and 66.7% for DeepSeek-R1. Because only six image-based items were available, these subgroup findings were considered exploratory and do not permit firm conclusions regarding radiographic interpretation. No significant temporal trend was identified in the exploratory model-based performance index.

Conclusions: GPT-5.1 achieved the highest accuracy on this publicly accessible DUS benchmark and significantly outperformed Claude Sonnet 4.5, Gemini 2.0 Flash, and DeepSeek-R1. However, the public availability of the questions and answer keys introduces a risk of benchmark contamination, and the very small image-based subgroup precludes generalisation regarding radiographic interpretation. These findings should therefore be interpreted as comparative performance on a public examination benchmark rather than evidence of clinical diagnostic competence.

## Introduction

Over the past two decades, advances in computing power and the growth of data have accelerated the integration of artificial intelligence (AI) into dentistry for image analysis, lesion detection, classification, and clinical workflow support [1-4]. Vision-based and multimodal systems have also been evaluated on dental radiographs and other oral images [5,6]. In parallel, large language models (LLMs) have become accessible tools for explanation generation, question answering, and educational support [7,8].

Professional examinations have therefore become a common benchmarking strategy for LLMs. Systematic evidence from national licensing examinations and prospective radiology-board evaluations shows strong but variable performance across model generations and task formats [9,10]. In dentistry, Sismanoglu and Capan reported that ChatGPT-4.0 outperformed Gemini Advanced on questions from the Turkish Dental Specialisation Examination (DUS) [11], whereas an earlier oral and maxillofacial radiology evaluation demonstrated more limited reliability for speciality-specific reasoning [12]. These findings indicate that examination performance cannot be generalised across models, specialities, or versions.

Dental-domain benchmarking has likewise shown model-dependent accuracy. Taşsöker compared multiple chatbots on oral radiology questions [13], while subsequent studies reported heterogeneous performance in oral pathology [14], periodontology [15], and prosthodontics [16]. Across these investigations, newer systems frequently performed better on knowledge-based multiple-choice items than on clinically contextualised or visually demanding tasks, underscoring the importance of evaluating both question type and speciality.

Language and assessment format also influence results. Sabaner and Yozgat demonstrated high but not identical Turkish- and English-language performance for ChatGPT-4o and Gemini 1.5 Pro on ophthalmology questions [17]. In UK dental assessments, Dave et al. found that model rankings differed according to multiple-choice versus short-answer formats [18]. A multidisciplinary bilingual DUS assessment similarly identified discipline-specific variation between Turkish and English questions [19]. Together, these observations support transparent reporting of language, prompt structure, model version, and interface conditions.

Oral and maxillofacial radiology is a particularly relevant domain for such evaluation because it combines factual knowledge with radiographic interpretation. The primary objective of the present study was to compare the overall answer accuracy of GPT-5.1, Claude Sonnet 4.5, Gemini 2.0 Flash, and DeepSeek-R1 on publicly accessible DUS oral and maxillofacial radiology questions. Secondary objectives were to explore differences according to theoretical versus image-based question type and to examine session-to-session variation in model performance across examination periods descriptively. These secondary analyses were considered exploratory and were not intended to assess the difficulty of intrinsic psychometric examination.

## Materials and methods

Study design and data source

This cross-sectional benchmark study used only publicly accessible examination materials and did not involve human participants, animals, or identifiable personal data; institutional ethics approval and informed consent were therefore not required. Multiple-choice questions (MCQs) were obtained from the oral and maxillofacial radiology component of the DUS, administered by the Student Selection and Placement Center (ÖSYM). The study was restricted to examination sets available in the official ÖSYM past-examination archive [20]. Published studies have likewise used the same or comparable publicly accessible ÖSYM/DUS question banks to benchmark LLMs in oral radiology and other dental specialities [11,13,15,16,19,21]. After screening the 2012-2021 examination sets against the official answer keys and excluding duplicates or invalid items, 132 questions were included: 126 theoretical and six image-based items. Each item contained five response options and one keyed answer.

Question categorisation

To explore differences in reasoning demands, questions were categorised as theoretical or image-based. Theoretical questions tested radiologic physics, radiation safety, dental anatomy, radiobiology, instrumentation and lesion classification. Image-based questions required interpretation of panoramic radiographs, periapical and occlusal films, cone beam computed tomography (CBCT) slices or clinical photographs. Questions were grouped by exam year, allowing us to detect potential trends in difficulty across the 2012-2021 timeframe. All questions were retained in their original Turkish to prevent translation bias, and ambiguous phrasing was resolved by consensus between two subject-matter experts.

LLMs and query procedure

The evaluated interfaces were GPT-5.1 (OpenAI, San Francisco, CA, USA), Claude Sonnet 4.5 (Anthropic, San Francisco, CA, USA), Gemini 2.0 Flash (Google DeepMind, Mountain View, CA, USA), and DeepSeek-R1 (DeepSeek, Hangzhou, China). Each question was submitted individually in a new chat using the same standardised instruction to return only the single best answer option, and previous-chat context was not carried forward. No application programming interface (API), locally hosted model, third-party wrapper, or external retrieval system was used. For image-based items, the image and the complete question stem with all five response options were submitted using the image-upload functionality available through the respective web interface during the testing period. For DeepSeek-R1, images were submitted through the DeepSeek web interface rather than through an external image-analysis, optical character recognition, or captioning system. No investigator-operated intermediary vision model was used. Because the internal preprocessing and model-routing architecture of the proprietary web interface was not exposed to the investigators, it was not possible to determine whether platform-level image preprocessing occurred before generation of the final DeepSeek-R1 response. Model outputs were recorded before scoring to preserve the original responses.

Sample size justification

The sample size was determined by a census of the eligible, publicly accessible DUS oral and maxillofacial radiology questions from 2012 to 2021; no a priori power calculation was performed because all questions meeting the predefined eligibility criteria were included. The final benchmark contained 132 paired observations, as every question was answered by each of the four models. Because only six image-based items were available, analyses by question type were considered exploratory and were interpreted cautiously.

Model versions, interface configuration, and access period

All model evaluations were conducted during a predefined testing period from November 1 to November 15, 2025, using the official browser-based web interfaces available at the time of testing. The model labels displayed by the interfaces were GPT-5.1, Claude Sonnet 4.5, Gemini 2.0 Flash, and DeepSeek-R1. No API access, third-party wrapper, locally deployed model, or browser extension was used. Each question was entered in a new conversation; previous-chat context was not retained, and the same standardised response instruction was used for all systems. Web-browsing functionality was disabled whenever this setting could be controlled, including for Gemini 2.0 Flash. Image-based questions were submitted through the native upload functionality available in the corresponding web interface, as described above.

The study log documented the common testing period and displayed model versions but did not retain model-specific or question-specific date and time stamps. Therefore, exact individual testing dates were not retrospectively reconstructed. These interface details are reported because model routing, platform updates, and proprietary web-interface configurations can change over time and may limit exact replication.

Scoring and reliability

Responses were scored as correct or incorrect against the official answer key. Two oral and maxillofacial radiologists independently verified transcription and scoring, and any discrepancy was resolved by consensus. This procedure was applied uniformly across models and question types.

Exploratory secondary analysis: model-based performance index and temporal variation

As an exploratory secondary analysis, session-to-session variation in model performance was descriptively assessed across the available DUS examination periods. For this purpose, a model-based difficulty proxy was calculated from the mean accuracy of Claude Sonnet 4.5, Gemini 2.0 Flash, and DeepSeek-R1.

GPT-5.1 was excluded from this calculation because it achieved 100% accuracy across all examination periods and therefore contributed no between-period variation. The proxy was calculated as follows:

$\text{Model-Based Difficulty Proxy (\%)} = 100 - \left[ \text{Mean accuracy (\%) of Claude Sonnet 4.5, Gemini 2.0 Flash, and DeepSeek-R1} \right]$

Higher values therefore indicate examination sessions in which these three models collectively achieved lower accuracy. Importantly, this measure was not intended to represent intrinsic or psychometrically validated examination difficulty. It is a model-dependent descriptive proxy and may reflect differences in model knowledge, reasoning, benchmark exposure, question composition, and other model-specific factors. In the absence of human candidate performance or item-response data, no inference regarding the true difficulty of individual DUS examinations was made. Temporal variation in this model-based proxy across the 13 examination periods was explored using linear regression, Spearman rank correlation, and the Mann-Kendall trend test. These analyses were descriptive and exploratory.

Statistical analysis

Correct-answer counts, accuracy percentages, and 95% Wilson confidence intervals (CIs) were calculated for each model. Fisher’s exact test compared theoretical and image-based items within each model. Because every question was answered by all four models, overall accuracy was compared using Cochran’s Q test; when the omnibus test was significant, two-sided pairwise McNemar tests were performed with a Bonferroni-adjusted significance threshold of α = 0.05/6 = 0.0083. As an exploratory secondary analysis, temporal variation in the model-based difficulty proxy was examined using linear regression, Spearman rank correlation, and the Mann-Kendall trend test. This proxy was not treated as a validated measure of intrinsic examination difficulty. Analyses were performed using IBM SPSS Statistics for Windows, Version 29 (Released 2022; IBM Corp., Armonk, New York, United States), and all tests were two-sided.

## Results

All 132 questions were evaluated by each model. GPT-5.1 achieved the highest overall accuracy, followed by Claude Sonnet 4.5, Gemini 2.0 Flash, and DeepSeek-R1. The distribution of correct and incorrect responses across the original DUS question-number positions represented in the eligible dataset is summarised in Table 1.

**Table 1:** Distribution of model responses by original DUS question-number position. The question-number categories shown are the positions represented by the 132 eligible oral and maxillofacial radiology items identified during screening of the publicly accessible 2012-2021 DUS examinations. They are presented to show how correct and incorrect responses were distributed across the original examination positions; no questions were purposively selected for display beyond the eligibility criteria. DUS: Turkish Dental Specialisation Exam

DUS question#	Claude, n (%)	Gemini, n (%)	GPT-5.1, n (%)	DeepSeek, n (%)
Question #30				
Wrong	0 (0.0)	0 (0.0)	0 (0.0)	0 (0.0)
Correct	2 (100.0)	2 (100.0)	2 (100.0)	2 (100.0)
Question #31				
Wrong	1 (7.7)	1 (7.7)	0 (0.0)	2 (15.4)
Correct	12 (92.3)	12 (92.3)	13 (100.0)	11 (84.6)
Question #32				
Wrong	0 (0.0)	0 (0.0)	0 (0.0)	2 (15.4)
Correct	13 (100.0)	13 (100.0)	13 (100.0)	11 (84.6)
Question #33				
Wrong	2 (15.4)	2 (15.4)	0 (0.0)	2 (15.4)
Correct	11 (84.6)	11 (84.6)	13 (100.0)	11 (84.6)
Question #34				
Wrong	0 (0.0)	1 (7.7)	0 (0.0)	1 (7.7)
Correct	13 (100.0)	12 (92.3)	13 (100.0)	12 (92.3)
Question #35				
Wrong	0 (0.0)	3 (23.1)	0 (0.0)	3 (23.1)
Correct	13 (100.0)	10 (76.9)	13 (100.0)	10 (76.9)
Question #36				
Wrong	2 (15.4)	4 (30.8)	0 (0.0)	4 (30.8)
Correct	11 (84.6)	9 (69.2)	13 (100.0)	9 (69.2)
Question #37				
Wrong	3 (23.1)	4 (30.8)	0 (0.0)	3 (23.1)
Correct	10 (76.9)	9 (69.2)	13 (100.0)	10 (76.9)
Question #38				
Wrong	3 (23.1)	3 (23.1)	0 (0.0)	2 (15.4)
Correct	10 (76.9)	10 (76.9)	13 (100.0)	11 (84.6)
Question #39				
Wrong	1 (7.7)	2 (15.4)	0 (0.0)	1 (7.7)
Correct	12 (92.3)	11 (84.6)	13 (100.0)	12 (92.3)
Question #40				
Wrong	1 (7.7)	1 (7.7)	0 (0.0)	4 (30.8)
Correct	12 (92.3)	12 (92.3)	13 (100.0)	9 (69.2)
Total				
Wrong	13 (9.8)	21 (15.9)	0 (0.0)	24 (18.2)
Correct	119 (90.2)	111 (84.1)	132 (100.0)	108 (81.8)

GPT-5.1 answered 132/132 questions correctly (100.0%; 95% CI, 97.2-100.0), compared with 119/132 for Claude Sonnet 4.5 (90.2%; 95% CI, 83.9-94.2), 111/132 for Gemini 2.0 Flash (84.1%; 95% CI, 76.9-89.4), and 108/132 for DeepSeek-R1 (81.8%; 95% CI, 74.4-87.5). Overall performance differed significantly among the four models (Cochran’s Q = 34.5; p < 0.001).

Table 2 compares model accuracy according to question type. Claude Sonnet 4.5, Gemini 2.0 Flash, and DeepSeek-R1 showed numerically lower accuracy on image-based items than theoretical items, whereas GPT-5.1 answered all items correctly. The largest observed difference was for Gemini 2.0 Flash (85.7% vs 50.0%; Fisher’s exact p = 0.051), but none of the within-model comparisons reached p < 0.05. Because the image-based subgroup contained only six items, these estimates were considered exploratory.

**Table 2:** Accuracy of the four large language models according to question type. p-values were calculated using Fisher’s exact test; - indicates not estimable because both question-type groups had 100% accuracy.

Model	Outcome	Theoretical, n (%)	Image-based, n (%)	p-value
Claude 4.5	Incorrect	12 (9.5)	1 (16.7)	0.470
	Correct	114 (90.5)	5 (83.3)	
Gemini 2.0	Incorrect	18 (14.3)	3 (50.0)	0.051
	Correct	108 (85.7)	3 (50.0)	
GPT-5.1	Incorrect	0 (0.0)	0 (0.0)	-
	Correct	126 (100.0)	6 (100.0)	
DeepSeek-R1	Incorrect	22 (17.5)	2 (33.3)	0.299
	Correct	104 (82.5)	4 (66.7)	

**Figure 1:**
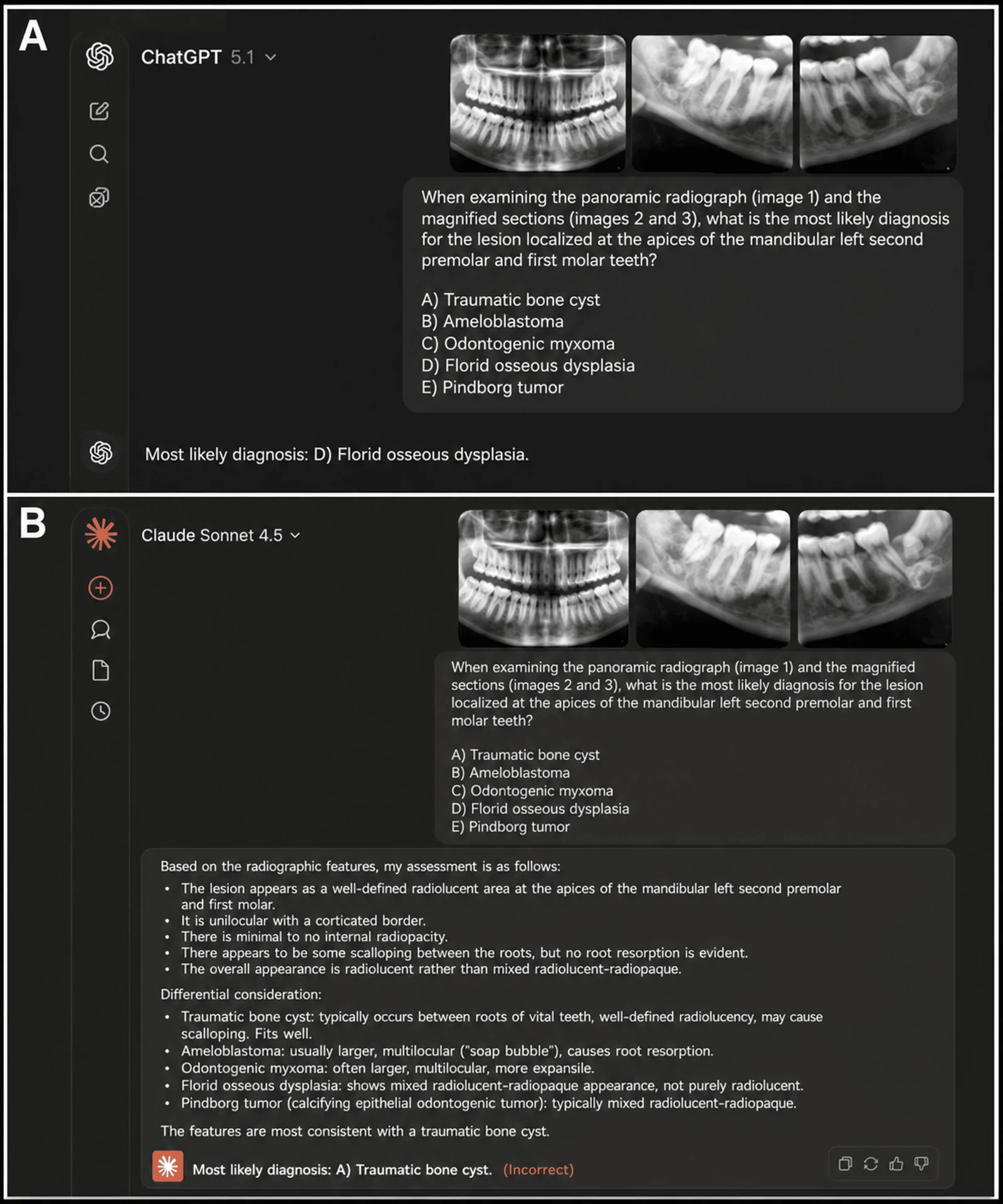
Example responses to an image-based mandibular lesion question. (A) GPT-5.1 selected the keyed answer, florid osseous dysplasia. (B) Claude Sonnet 4.5 selected an incorrect diagnosis. Original interface excerpts were cropped and enlarged for legibility; response content was not altered.

Figure 1 illustrates the multimodal query format for one image-based mandibular lesion item. Panel A shows the GPT-5.1 response, which selected the keyed diagnosis, whereas Panel B shows the Claude Sonnet 4.5 response to the same case, which selected an incorrect diagnosis. To improve legibility while preserving the original model outputs, the interface screenshots were cropped to the question and answer regions and enlarged without changing their content.

Cochran’s Q test demonstrated a statistically significant overall difference in accuracy among the four models (Q = 34.5; p < 0.001). Pairwise McNemar tests showed that GPT-5.1 outperformed Claude Sonnet 4.5 (p = 0.0002), Gemini 2.0 Flash (p < 0.0001), and DeepSeek-R1 (p < 0.0001) at the Bonferroni-adjusted threshold of 0.0083. Differences among Claude Sonnet 4.5, Gemini 2.0 Flash, and DeepSeek-R1 were not significant after adjustment.

**Figure 2:**
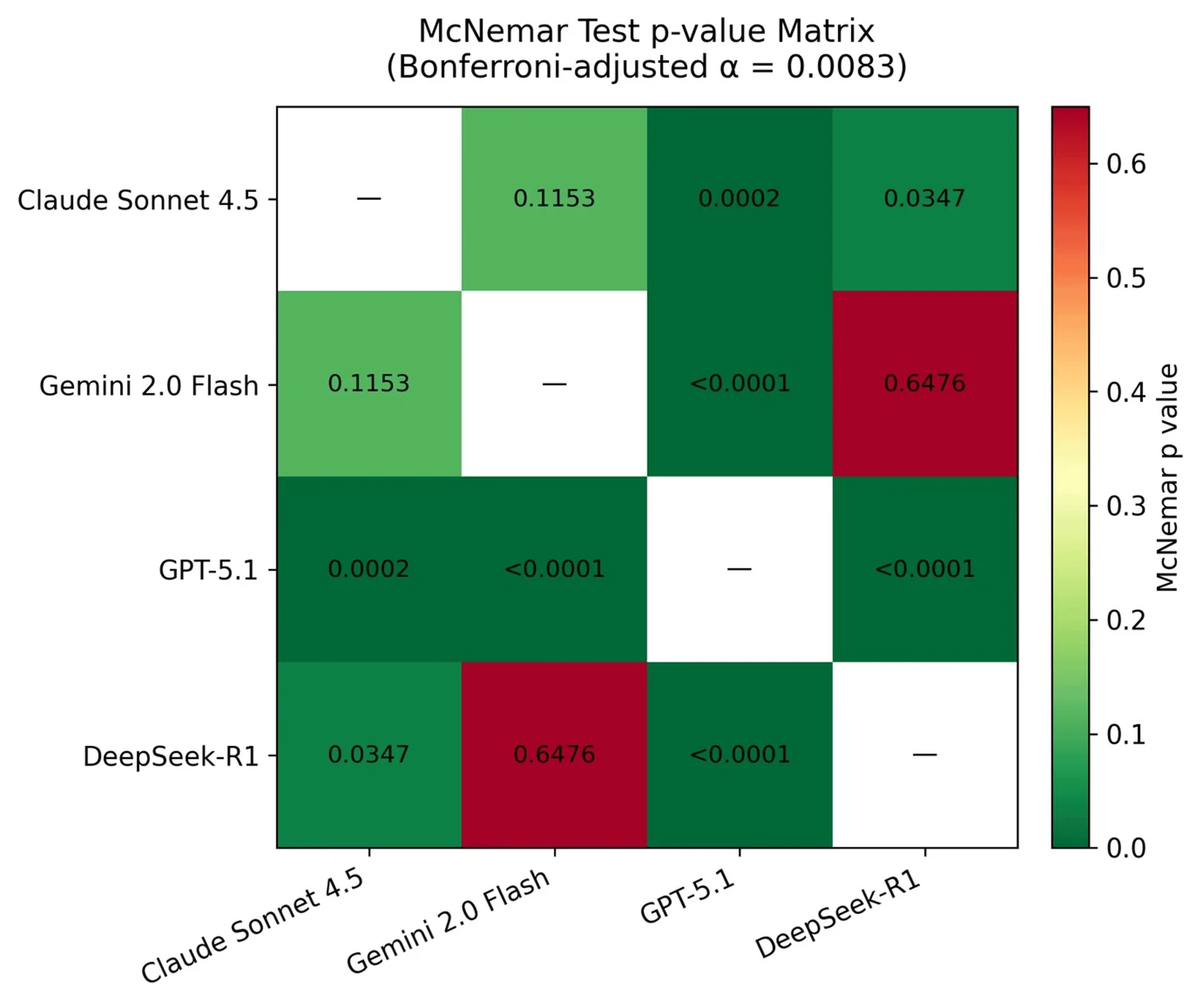
Pairwise McNemar p-value matrix. The Bonferroni-adjusted significance threshold was α = 0.0083.

Figure 2 presents the pairwise McNemar p-value matrix together with the Bonferroni-adjusted significance threshold (α = 0.0083). The low p-values for comparisons involving GPT-5.1 visually reinforce the statistically significant pairwise differences, whereas the comparisons among Claude Sonnet 4.5, Gemini 2.0 Flash, and DeepSeek-R1 remain above the adjusted threshold.

As an exploratory secondary analysis, a model-based difficulty proxy was calculated to describe session-to-session variation in the collective performance of Claude Sonnet 4.5, Gemini 2.0 Flash, and DeepSeek-R1. GPT-5.1 was excluded from this calculation because it achieved 100% accuracy in every examination period and therefore provided no between-period variability. The proxy was calculated as:

$\text{Model-Based Difficulty Proxy (\%)} = 100 - \left( \text{Mean Accuracy of Claude Sonnet 4.5, Gemini 2.0 Flash, and DeepSeek-R1} \right)$

**Figure 3:**
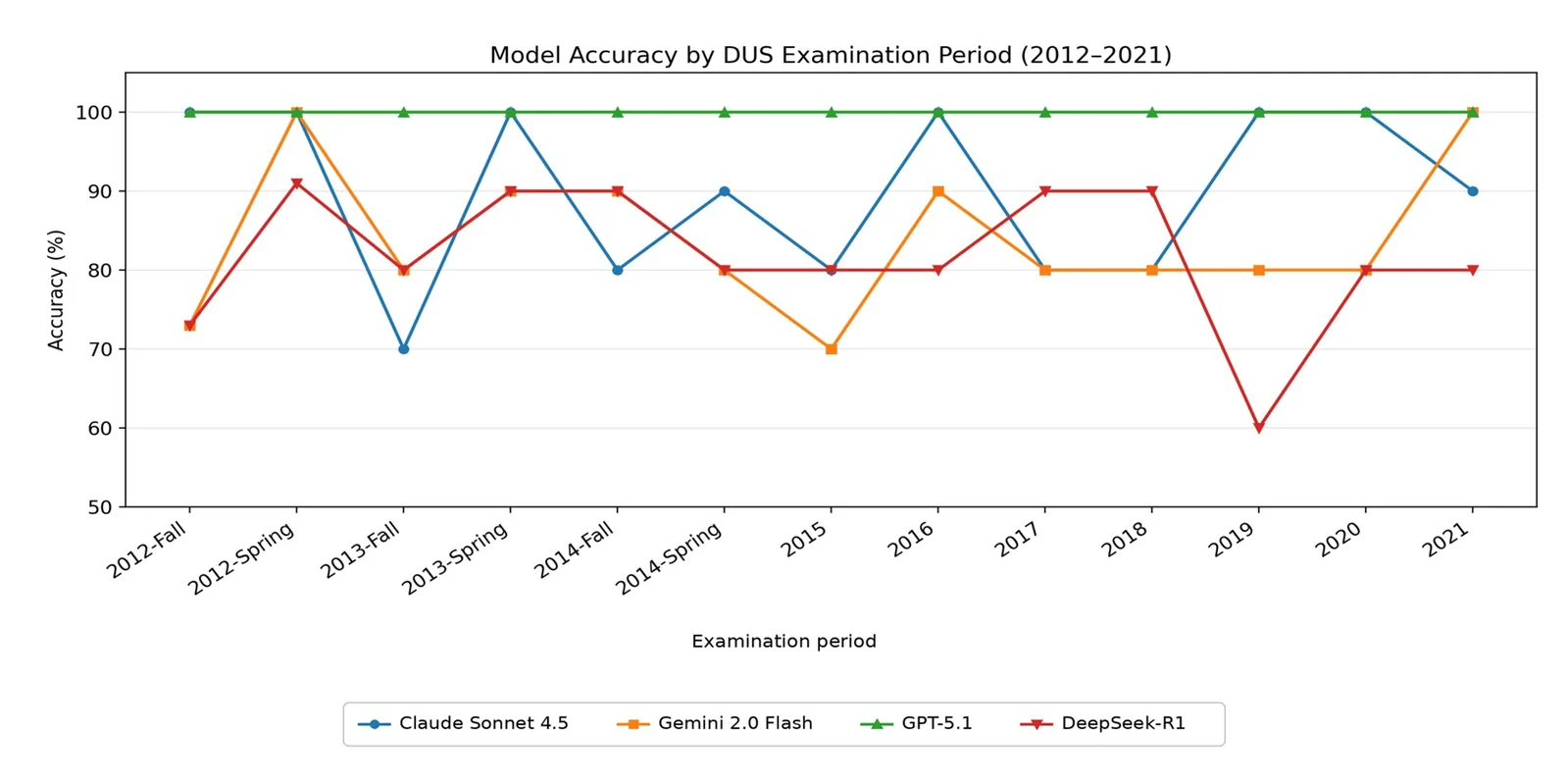
Accuracy of the four large language models across DUS oral and maxillofacial radiology examination periods (2012-2021). GPT-5.1 remained at 100% across all periods, whereas Claude Sonnet 4.5, Gemini 2.0 Flash, and DeepSeek-R1 showed greater session-to-session variability. DUS: Turkish Dental Specialisation Exam

Higher values indicate examination periods in which these three models collectively achieved lower accuracy. This measure should not be interpreted as a psychometrically validated estimate of intrinsic DUS examination difficulty. No statistically significant increasing or decreasing temporal pattern was identified across the 13 examination periods from 2012 to 2021 (linear regression p = 0.828; Spearman p = 0.943; Mann-Kendall p = 1.000). Figure 3 shows examination-period accuracy for all four models and demonstrates that GPT-5.1 remained at 100%, whereas the other systems showed greater session-to-session variation.

**Figure 4:**
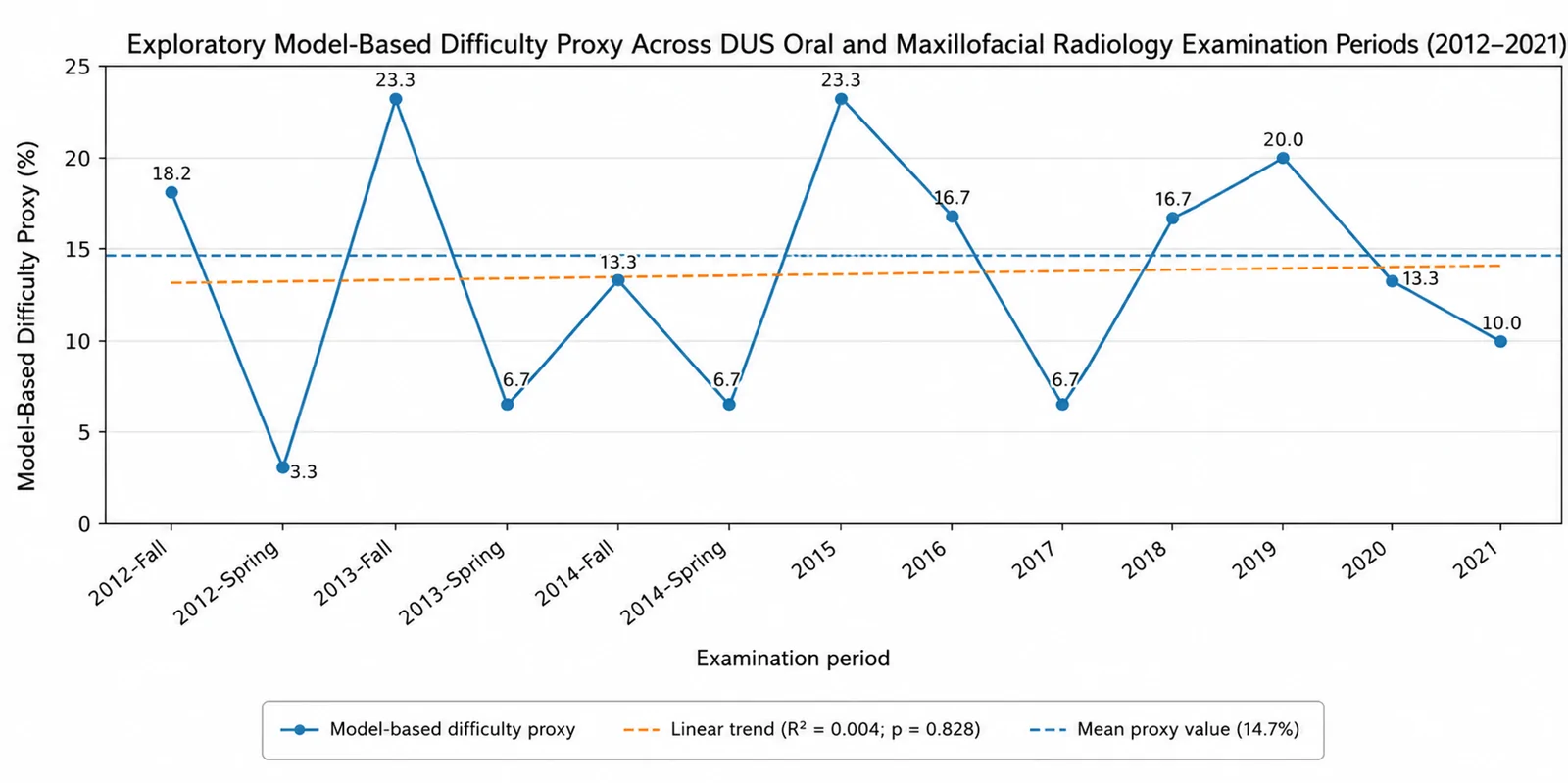
Exploratory model-based difficulty proxy across DUS oral and maxillofacial radiology examination periods from 2012 to 2021. The proxy was calculated as 100% minus the mean accuracy of Claude Sonnet 4.5, Gemini 2.0 Flash, and DeepSeek-R1. Higher values indicate lower collective model accuracy and should not be interpreted as psychometrically validated measures of intrinsic examination difficulty. DUS: Turkish Dental Specialisation Exam

Figure 4 plots the difficulty index for each DUS examination period, calculated as 100% minus the mean accuracy of Claude Sonnet 4.5, Gemini 2.0 Flash, and DeepSeek-R1. The highest index values occurred in Fall 2013 and 2015, whereas Spring 2012 had the lowest value. The fitted trend line was nearly flat, consistent with the non-significant time-trend analyses.

**Figure 5:**
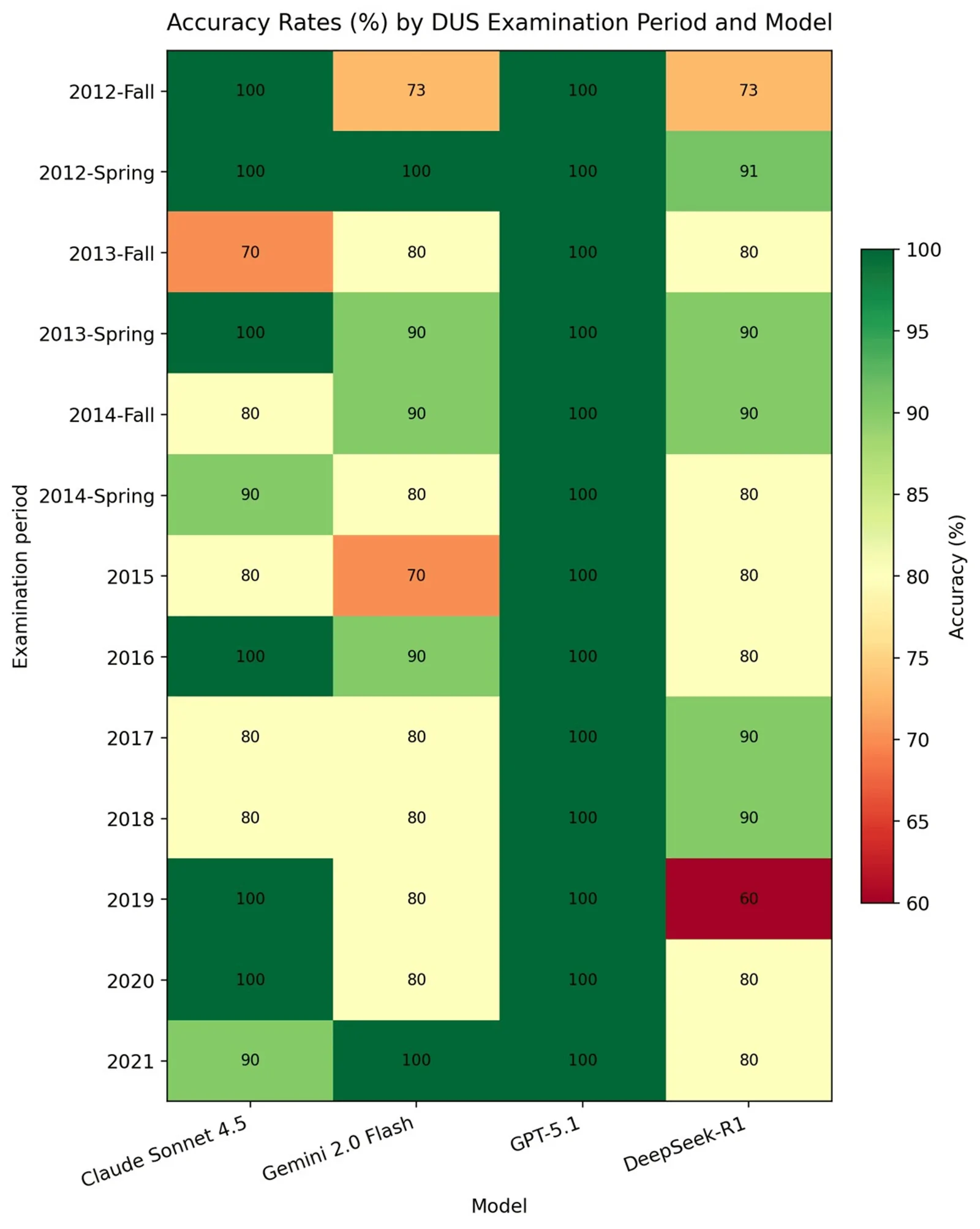
Accuracy heatmap by DUS examination period and model. Rows represent examination periods and columns represent Claude Sonnet 4.5, Gemini 2.0 Flash, GPT-5.1, and DeepSeek-R1. DUS: Turkish Dental Specialisation Exam

Figure 5 summarises model accuracy across the 13 examination periods as a heatmap. The cell values permit direct comparison of each model by exam session and show the consistent 100% performance of GPT-5.1 together with greater variability for Claude Sonnet 4.5, Gemini 2.0 Flash, and DeepSeek-R1.

## Discussion

In this benchmark of 132 publicly accessible DUS oral and maxillofacial radiology questions, GPT-5.1 achieved the highest observed accuracy and significantly outperformed the other three interfaces. Claude Sonnet 4.5 ranked second, followed by Gemini 2.0 Flash and DeepSeek-R1, although the differences among these three models were not significant after correction for multiple testing. The result should be interpreted as performance on a public examination benchmark rather than as evidence of specialist-level clinical competence.

Recent oral and maxillofacial radiology studies provide useful context for these findings. Icen and Temur evaluated 200 DUS oral and maxillofacial radiology questions from 2012 to 2025 and found significant performance differences among contemporary systems, with ChatGPT-5.2 showing the highest accuracy [21]. Haylaz et al. likewise reported substantial variation among four LLMs on 240 oral and maxillofacial radiology questions, including a dedicated image-based subset [22]. Earlier DUS and dental-speciality studies also documented model-dependent rankings in general dental assessment, oral radiology, oral pathology, periodontology, prosthodontics, and bilingual testing [11,13-16,19].

The image-based subgroup in the present study requires particular caution. Prior dentomaxillofacial radiology research and broader reviews indicate that visual performance is less stable than text-only performance and is sensitive to image quality, platform preprocessing, and the way clinical information is supplied [6,12,22-24]. Atakır et al. showed that ChatGPT-4o diagnostic accuracy increased markedly when clinical information and answer options accompanied the images [23]. In our study, each image was likewise presented with the complete question stem and five response options, so the observed result may reflect a combination of image interpretation, textual reasoning, and multiple-choice test-taking cues.

The image-based subgroup in the present study requires particular caution. Only six image-based questions were publicly available during the study period, resulting in very imprecise estimates and preventing robust conclusions regarding multimodal diagnostic performance. Although GPT-5.1 answered all six image-based questions correctly, this result should not be interpreted as evidence of consistently superior radiographic interpretation. Likewise, the numerically lower image-based accuracy observed for Claude Sonnet 4.5, Gemini 2.0 Flash, and DeepSeek-R1 cannot establish a systematic disadvantage because of the very small number of observations. The findings should therefore be considered hypothesis-generating and require confirmation using larger, balanced, prospectively assembled radiographic datasets [6,22-24].

Text-based examination performance also varies according to the benchmark and model generation. Taşsöker and Akyüz evaluated 123 text-only oral radiology questions from the DUS bank and reported the highest accuracy for ChatGPT-4 Omni [13]. Sismanoglu and Capan [11], Kandemir and Sarıbaş [15], Tosun and Yılmaz [16], Dündar Sarı and Sezer [19], and Icen and Temur [21] similarly demonstrated that rankings change across specialities, languages, and model generations. These differences emphasise that results obtained with one interface at one time point should not be extrapolated uncritically to later versions.

Language and response format are additional sources of variability. Sabaner and Yozgat found very high performance for both Turkish and English ophthalmology questions but small language-dependent differences between systems [17]. In UK dentistry and dental hygiene and therapy assessments, Dave et al. reported that relative performance depended on whether questions were multiple-choice or short-answer [18]. The bilingual DUS analysis by Dündar Sarı and Sezer also demonstrated discipline-specific language effects [19]. These findings support documenting the original question language, prompt wording, interface, and access period in reproducible LLM benchmarking.

A major concern when interpreting the present findings is benchmark contamination. The DUS questions and their answer keys were publicly accessible before model testing, and the extent to which individual items, answer keys, or derivative educational materials may have been represented in model training corpora or other platform-accessible data cannot be determined. Consequently, the contribution of prior exposure to the observed performance cannot be quantified. This issue is particularly relevant to the flawless GPT-5.1 result. The present study cannot determine whether the 100% accuracy of GPT-5.1 resulted from superior reasoning, greater domain knowledge, prior exposure to the benchmark, memorisation of specific questions or answer keys, or a combination of these factors. There is no direct evidence that permits attribution of its perfect score to benchmark contamination, but contamination also cannot be excluded. Accordingly, the result should be interpreted strictly as performance on a publicly accessible benchmark rather than as independent evidence of specialist-level reasoning or clinical competence.

Written-examination performance should also not be equated with educational equivalence to human learners. Ullah et al. directly compared ChatGPT with dental students in a dental anatomy examination and highlighted differences between AI and student performance [25]. Kavadella et al. found that real-life implementation of ChatGPT can offer practical value in undergraduate dental education while still requiring structured guidance and critical appraisal [26]. Accordingly, LLMs are better positioned as supervised adjuncts for question practice, explanation generation, and formative feedback than as replacements for conventional teaching or expert assessment.

Safety and responsible use remain central to educational integration. LLMs may generate fluent but incorrect statements, and hallucination remains a clinically relevant limitation that requires verification against authoritative sources [27]. Ethical integration into dental curricula should therefore address transparency, bias, privacy, accountability, and the limits of automated advice rather than focusing only on technical performance [28]. These principles are especially important when systems are used in high-stakes assessment or image-based clinical learning.

Student attitudes indicate a parallel need for formal AI education. Keser and Namdar Pekiner reported that dental students were generally receptive to AI-assisted radiological diagnosis but that familiarity and knowledge were not universal [29]. Taşsöker and Akyüz similarly found predominantly positive attitudes toward AI in oral radiology and substantial support for incorporating AI education into dental training [30]. Together, these studies suggest that enthusiasm should be matched with structured instruction in verification, limitations, and responsible use.

From an educational perspective, the strong theoretical-question performance observed in the present study supports carefully supervised use of LLMs for revision and formative assessment. Their outputs should nevertheless be checked against validated textbooks, guidelines, and expert interpretation, particularly for radiographic cases. Future educational studies should evaluate not only answer selection but also explanation quality, calibration, consistency across repeated runs, and the effect of AI assistance on learner reasoning and retention [7,8,26-28].

The exploratory model-based difficulty proxy also warrants cautious interpretation. Because it was derived entirely from LLM accuracy, it does not provide an independent estimate of examination difficulty and should not be considered equivalent to a conventional psychometric difficulty index derived from human examinee responses. Session-to-session variation may reflect question composition, model-specific knowledge gaps, benchmark exposure, or other characteristics of the evaluated systems. The absence of a temporal trend therefore indicates only that the collective performance of the three included models did not change systematically across the examined sessions; it does not demonstrate that the intrinsic difficulty of DUS remained stable over time.

Several limitations should be acknowledged. The dataset originated from a single national examination source, and only six questions were image-based, substantially limiting conclusions regarding radiographic interpretation. The questions and answer keys were publicly accessible before testing, creating an unavoidable risk of benchmark contamination that cannot be quantified from the present design. The study used proprietary browser-based interfaces whose internal model routing and image-preprocessing procedures were not fully observable and may change over time. Although the common testing period and displayed model versions were recorded, model-specific and question-specific timestamps were not retained. The study also used one recorded response per item and did not include a contemporaneous human comparator. Finally, the exploratory model-based difficulty proxy was derived from LLM performance and does not represent a psychometrically validated estimate of intrinsic examination difficulty. Future studies should use access-controlled or prospectively developed questions, larger and more balanced image datasets, repeated model runs, preregistered prompts and scoring rules, fixed model snapshots where available, and direct comparisons with students and specialists.

## Conclusions

GPT-5.1 achieved the highest accuracy on publicly accessible DUS oral and maxillofacial radiology questions and significantly outperformed Claude Sonnet 4.5, Gemini 2.0 Flash, and DeepSeek-R1. However, the public availability of the benchmark creates an unquantifiable risk of prior model exposure, and only six image-based questions were available. Accordingly, the findings demonstrate comparative performance on this specific public examination benchmark but should not be interpreted as evidence of clinical diagnostic competence or consistently superior radiographic interpretation. Larger, access-controlled, prospectively designed, and externally validated studies are required before such systems can be considered for high-stakes assessment or radiographic decision-making.
